# Sternal tumors surgery: extent of resection, type of reconstruction and survival

**DOI:** 10.1186/1749-8090-8-S1-O264

**Published:** 2013-09-11

**Authors:** D Petrov, Tz Minchev, G Iankov, E Goranov, Sv Alexov

**Affiliations:** 1 Thoracic Surgery Department, Saint Sophia University Hospital of Pulmonary Diseases, Medical University-Sofia, Bulgaria; 2 Thoracic Surgery Ward, Tokuda Hospital, Sofia, Bulgaria

## Background

Objective was to evaluate the postoperative results after different sternal resections for malignances.

## Methods

A total of 32 patients (11 males, 21 females, mean age 51.9 years) were operated on during a 7 years period in two institutions. There were 16 sarcomas (9 chondrosarcomas, 3 radiation-induced sarcomas, 2 Ewing sarcoma, 2 osteosarcoma), 1 recurrent desmoid tumor, 3 local breast cancer recurrences, 1 plasmocytoma, and 11 metastatic tumors (5 from breast cancer, 2 rabdomyosarcoma, 2 liposarcoma, 1 NSCLC, 1 prostate cancer). Four-five cm free margins on each side were achieved in all patients with total sternectomy (13), subtotal sternal resection (17), and partial resection (2). Concurrent en bloc resections included anterior ribs (7), clavicle (6), pericardium (2), brachiocephalic vein (2), and diaphragm (1). The chest was reconstructed with Gore-tex (5) or Marlex mesh (22) and myocutaneous flap in 18 (56.25%) patients or omentum in 6 (18.75%) patients. Only double layer Marlex mesh with breast mobilization was performed in 5 women (15.62%). Combination of prostetic material and metal bars or wires was needed in 6 patients after total sternectomy (18.75%). Multimodality treatment pre- or postoperatively was administered in most of the patients.

## Results

There was no 30-day operative mortality. Mechanical ventilation for 5 days was needed in a 78 years old female after total sternectomy. Local suppuration was found in 3 patients. The mean in-hospital stay was 12.7 days. After a median follow-up of 56 months the overall 5-year survival was 58%, with a median survival of 61 months. Local recurrence occurred in 3 patients, who underwent a repeat total resection. Metastases developed in 2 patients.

## Conclusion

Wide sternal resection (4-5 cm free margins) is a safe and effective treatment of sternal malignances. The chest wall reconstruction depends on the size and site of the resection, and should be planned with plastic surgeons.

